# Novel potential predictive markers of sunitinib outcomes in long-term responders versus primary refractory patients with metastatic clear-cell renal cell carcinoma

**DOI:** 10.18632/oncotarget.16494

**Published:** 2017-03-23

**Authors:** Javier Puente, Nuria Laínez, Marta Dueñas, María José Méndez-Vidal, Emilio Esteban, Daniel Castellano, Mónica Martinez-Fernández, Laura Basterretxea, María José Juan-Fita, Luis Antón, Luis León, Julio Lambea, Begoña Pérez-Valderrama, Sergio Vázquez, Cristina Suarez, Xavier Garcia del Muro, Enrique Gallardo, José Pablo Maroto, M Luz Samaniego, Beatriz Suárez-Paniagua, Julián Sanz, Jesús M. Paramio, SOGUG (Spanish Oncology Genitourinary Group)

**Affiliations:** ^1^ Medical Oncology Department, Instituto de Investigación Biomédica, Hospital Clínico Universitario San Carlos, Madrid, Spain; ^2^ Medical Oncology Department, Complejo Hospitalario de Navarra, Pamplona, Spain; ^3^ Molecular Oncology Unit CIEMAT and Instituto Investigación Biomédica, Hospital Universitario 12 de Octubre, Madrid, Spain; ^4^ CIBERONC, Spain; ^5^ Medical Oncology Department, Hospital Universitario Reina Sofía, Córdoba, Spain; ^6^ Medical Oncology Department, Hospital Universitario Central de Asturias, Oviedo, Spain; ^7^ Medical Oncology Department, and Instituto Investigación Biomédica, Hospital Universitario 12 de Octubre, Madrid, Spain; ^8^ Medical Oncology Department, Hospital Donostia, Donostia, Spain; ^9^ Medical Oncology Department, Instituto Valenciano de Oncología, Valencia, Spain; ^10^ Medical Oncology Department, Complejo Hospitalario Universitario de A Coruña, A Coruña, Spain; ^11^ Promoción e Planificación da Investigación Sanitaria, Axencia de Coñecemento en Saúde, Santiago de Compostela, Spain; ^12^ Medical Oncology Department, Hospital Clínico de Zaragoza, Zaragoza, Spain; ^13^ Medical Oncology Department, Hospital Universitario Virgen del Rocío, Sevilla, Spain; ^14^ Medical Oncology Department, Hospital Universitario Lucus Augusti, Lugo, Spain; ^15^ Vall d'Hebron Institute of Oncology, Hospital Universitari Vall d' Hebron, Universitat Autònoma de Barcelona, Barcelona, Spain; ^16^ Medical Oncology Department, Institut Català d'Oncologia, Hospital Duran i Reynals, L'Hospitalet, Barcelona, Spain; ^17^ Medical Oncology Department, Hospital Universitari Parc Taulí, Sabadell, Spain; ^18^ Medical Oncology Department, Hospital de la Santa Creu i Sant Pau, Barcelona, Spain; ^19^ Statistical Department, Trial Form Support TFS people, Madrid, Spain; ^20^ Oncology Medical Department, Trial Form Support, Madrid, Spain; ^21^ Pathology Department, Hospital Clínico Universitario San Carlos, Madrid, Spain

**Keywords:** sunitinib, metastatic renal cell carcinoma, biomarkers, long-term responders, primary refractory

## Abstract

**Background:**

Several potential predictive markers of efficacy of targeted agents in patients with metastatic renal cell carcinoma (mRCC) have been identified. Interindividual heterogeneity warrants further investigation.

**Patients and methods:**

Multicenter, observational, retrospective study in patients with clear-cell mRCC treated with sunitinib. Patients were classified in two groups: long-term responders (LR) (progression-free survival (PFS)≥22 months and at least stable disease), and primary refractory (PR) (progressive disease within 3-months of sunitinib onset). Objectives were to compare baseline clinical factors in both populations and to correlate tumor expression of selected signaling pathways components with sunitinib PFS.

**Results:**

123 patients were analyzed (97 LR, 26 PR). In the LR cohort, overall response rate was 79% and median duration of best response was 30 months. Median PFS and overall survival were 43.2 (95% confidence intervals[CI]:37.2-49.3) and 63.5 months (95%CI:55.1-71.9), respectively. At baseline PR patients had a significantly lower proportion of nephrectomies, higher lactate dehydrogenase and platelets levels, lower hemoglobin, shorter time to and higher presence of metastases, and increased Fuhrman grade. Higher levels of *HEYL*, *HEY* and *HES1* were observed in LR, although only *HEYL* discriminated populations significantly (AUC[ROC]=0.704; cut-off=34.85). Increased levels of hsa-miR-27b, hsa-miR-23b and hsa-miR-628-5p were also associated with prolonged survival. No statistical significant associations between hsa-miR-23b or hsa-miR-27b and the expression of c-Met were found.

**Conclusions:**

Certain mRCC patients treated with sunitinib achieve extremely long-term responses. Favorable baseline hematology values and longer time to metastasis may predict longer PFS. *HEYL*, hsa-miR-27b, hsa-miR-23b and hsa-miR-628-5p could be potentially used as biomarkers of sunitinib response.

## INTRODUCTION

Over the past years multiple molecularly targeted agents for the treatment of advanced renal cell carcinoma (RCC) have been developed, greatly improving clinical benefit to patients [1].

Sunitinib, a multitargeted tyrosine kinase inhibitor, was approved worldwide as a first-line treatment for selected clear-cell metastatic RCC (mRCC) patients. Sunitinib efficacy was established in a pivotal Phase 3 study comparing sunitinib and interferon-α as first-line treatment in 750 patients with mRCC, showing a significantly higher objective response ratio (47% vs 12%, respectively; *P*<0.001), significantly longer progression-free survival (PFS) (median: 11 vs 5 months; Hazard ratio[HR]=0.539; 95% confidence intervals [CI]:0.451, 0.643; *P*<0.001), and longer overall survival (OS) (median: 26.4 vs 21.8 months; HR=0.82; 95%CI:0.67, 1.00, *P*=0.051) in the sunitinib group [2]. However, 9-21% of patients treated with sunitinib exhibit progressive disease as best response.

Similar to other conventional and molecular-targeted antitumor agents, there is an interindividual variability in response to sunitinib treatment. Potential serum-, radiological-, clinical- and tissue-based predictive biomarkers have been investigated across multiple agents [3–5], obtaining promising results. Additionally, certain adverse events (AEs) (i.e. Hypertension, Hypothyroidism) induced by sunitinib in mRCC patients may be associated with an improvement in clinical outcomes [6, 7].

Inherited genetic variability may be one of several contributing tumor- and host-related factors underlying individual treatment response. Specific signaling pathways such as Notch [8], Hedgehog [9] and Wingless (Wnt)-β-catenin [10] are critical for embryonic development, and for self-renewal and differentiation in adult stem cells, being also involved in tumor development and maintenance [11]. Remarkably, they are also involved in normal and pathological angiogenesis and, thus, have been considered as indicators of response to specific antiangiogenic therapies.

The microRNAs (miRNAs) are key regulators of oncogenic processes [12], representing a promising novel group of biomarkers. They are essential modulators of the above commented developmental signaling pathways, and are also involved in cancer stem cell maintenance [13].

The efficacy and safety of sunitinib in a subset of long-term responders has been previously described in retrospective pooled cohorts [14–16], although a comparison of extreme responders and non-responders is unpublished.

In the present analysis, we compared the baseline clinical characteristics and the levels of expression of various genes and miRNAs involved in Notch, Hedgehog, Wnt, hypoxia, epithelial mesenchymal transition and stem cell maintenance signaling in two extreme groups of patients: those with the highest benefit (long-term responders, LR) and those resistant to sunitinib (primary refractory, PR). We also explored the occurrence of several AEs as potential biomarkers of sunitinib efficacy.

## RESULTS

### Clinical characteristics

One hundred and twenty-three patients from 16 Spanish centers were included, of which 97 (79%) were LR and 26 (21%) were identified as PR. In the LR cohort, partial response was achieved in 59% of patients, complete response in 21% of patients and SD in 21%. Median duration of best response was 29 months and 33 months in partial and complete responders, respectively. At the time of analysis 51 patients had progressed. Median PFS was 43.2 months (95%CI: 37.2-49.3). Median OS was 63.5 months (95%CI: 55.1-71.9). In the PR cohort, median PFS and OS were 2.8 (95%CI: 2.5-3.1) and 7.0 months (95%CI: 3.3-10.7), respectively.

Baseline clinical characteristics that were significantly different between PR and LR patients are depicted in Table 1. In the overall population, 94% of patients had clear-cell histology with 7 patients having mixed histology (clear-cells plus sarcomatoid/papilar). Fourteen patients (11%) received sunitinib after cytokines and 109 patients (89%) in first-line, with no significant differences between groups.

**Table 1:** Demographic and clinical characteristics

Baseline patient characteristics		LR (n=97)	PR (n=26)	*P*-value
ECOG PS, *N* (%)	0	44 (55.0)	5 (23.8)	<0.01
	1	34 (42.5)	13 (61.9)	
	2	0 (0)	3 (14.3)	
Nephrectomy, *N* (%)	No	2 (2.1)	4 (15.4)	<0.01
	Yes	94 (97.9)	22 (84.6)	
LDH, UI/L, mean (95%CI)		258.4(235.9-281.0)	380.3(317.1-443.6)	<0.001
Hemoglobin, g/dl, mean (95%CI)		13.81(13.46-14.17)	11.96(11.07-12.86)	<0.001
Platelets, 10^3^/mm^3^, mean (95%CI)		240.6(223.6-257.6)	368.6(303.7-433.6)	<0.001
Time from primary to metastatic diagnosis, months, median (range)		24.7 (2.2 to 68.1)	2.5 (0.0 to 15.8)	<0.01
Time from diagnosis to treatment onset, months, median (range)		28.7 (6.8 to 71.9)	3.8 (1.2 to 23.0)	<0.01
Metastasis at diagnosis, *N* (%)	No	63 (69.2)	12 (46.2)	<0.05
	Yes	28 (30.8)	14 (53.8)	
Lung metastasis, *N* (%)		59 (60.8)	23 (88.5)	<0.01
Brain metastasis, *N* (%)		3 (3.1)	4 (15.4)	<0.05
Hepatic metastasis, *N* (%)		7 (7.2)	6 (23.1)	<0.05
Fuhrman grade	Grade 1	7 (10.3)	0 (0)	<0.05
	Grade 2	21 (30.9)	4 (23.5)	
	Grade 3	30 (44.1)	5 (29.4)	
	Grade 4	10 (14.7)	8 (47.1)	
Heng risk factors, *N* (%)	0	22 (23.7)	2 (8.7)	<0.01
	1	49 (52.7)	7 (30.4)	
	2	18 (19.4)	11 (47.8)	
	3	4 (4.3)	3 (13.0)	

ECOG-PS: Eastern Cooperative Oncology Group Performance Status; LR: long-term responders; PR: primary refractory; LDH: lactate dehydrogenase; CI: confidence intervals.

Sunitinib was administered once-daily at either a starting dose of 50 mg (93% of patients), 37.5 mg (5%) or 25 mg (2%) following 4-weeks-on/2-weeks-off schedule. The initial dose was reduced in 73% of patients (84% in LR group, 32% in PR group; *P*<0.001), mainly due to toxicity (92% of reductions). At the time of the study the alternative schedule 2 weeks-on/1 week-off was not established in clinical practice to manage toxicity.

Heng prognostic criteria were not significantly correlated with response in our population (*P*=0.109). However, most of the components individually, except for calcium and neutrophil levels, showed significant differences between both cohorts, and 61% of PR patients presented ≥2 Heng risk factors compared to 24% in LR group.

### Molecular assessment

**Figure 1:**
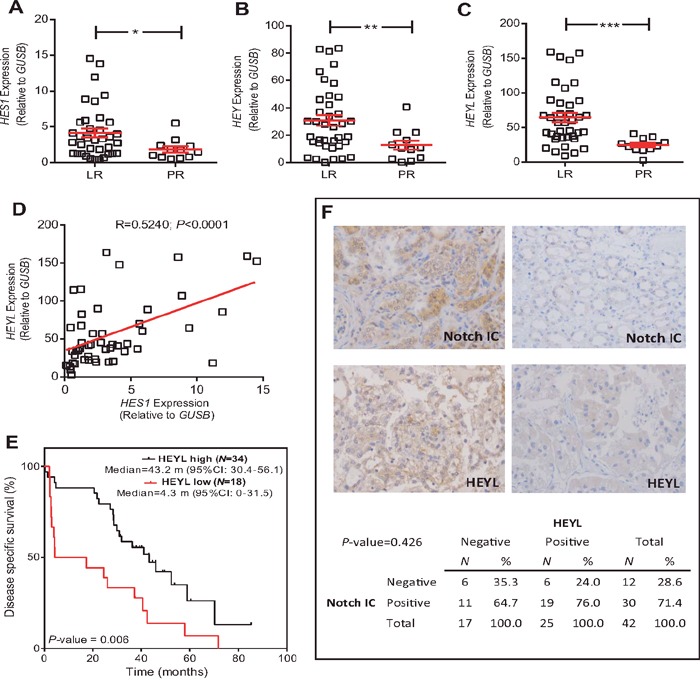
*HEYL* expression is associated with LR population **(A-C)** RTqPCR analyses showing the expression of *HES1* (A), *HEY* (B) and *HEYL* (C) genes in LR and PR populations. Statistical significance was obtained by Mann–Whitney's test *P-value ≤0.05; ** *P*-value ≤0.01, *** *P*-value ≤0.005. **(D)** Correlation between the *HES1* and *HEYL* expression values. *P*-value was estimated by Pearson correlation. **(E)** Kaplan-Meier analysis showing that high *HEYL* levels were associated with increased survival (*P*-value was obtained by the log-rank test). **(F)** Representative immunohistochemistry images Notch intracellular domain (NotchIC) and HEYL positive and negative tumors, and contingency tables showing the absence of significant association between NotchIC and HEYL. RTqPCR: Quantitative real-time reverse transcription polymerase chain reaction; LR: long-term responders; PR: primary refractory; CI: confidence intervals; m: months.

**Figure 2:**
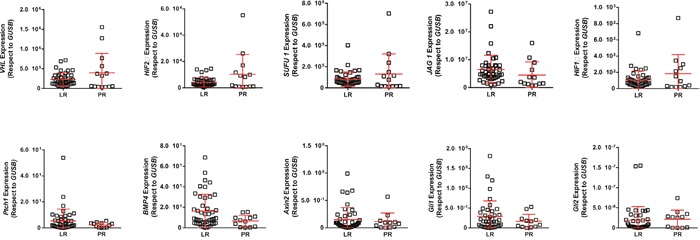
RTqPCR analyses showing the expression of quoted genes in LR and PR populations RTqPCR: Quantitative real-time reverse transcription polymerase chain reaction; LR: long-term responders; PR: primary refractory.

Fifty-two primary clear-cell primary tumor samples corresponding to 39 LR (75%) and 13 PR patients (25%) were analyzed. Among the different genes analyzed, only *HES1, HEY* and *HEYL*, putative effectors of Notch signaling, showed increased expression in the LR group compared to PR group (Figure 1A, 1B, and 1C, respectively; Figure 2). A significant positive internal correlation between the expression of these 3 genes was also observed (Figure 1D, and data not shown).

After the ROC analyses, only *HEYL* levels discriminated the studied populations with significant sensitivity and specificity (AUC=0.704). These analyses also provided a possible cut-off value of 34.85 (reference: *GUSB*). Of note, when the patients were classified according to their *HEYL* levels using this cut-off, an increased disease-specific survival in the high expression group was observed (Figure 1E).

Since *HEYL* is considered an effector of Notch signaling, we monitored whether LR and PR tumors displayed activated Notch. The expression of intracellular Notch domain (Notch-IC) and HEYL was determined in a TMA containing the previously analyzed samples. The samples were classified as negative or positive given their relatively small number (representative examples provided in Figure 1F). No statistically significant relationship between Notch-IC and HEYL positive staining was found (*P*=0.426) (Figure 1F).

### MicroRNA assessment

**Figure 3:**
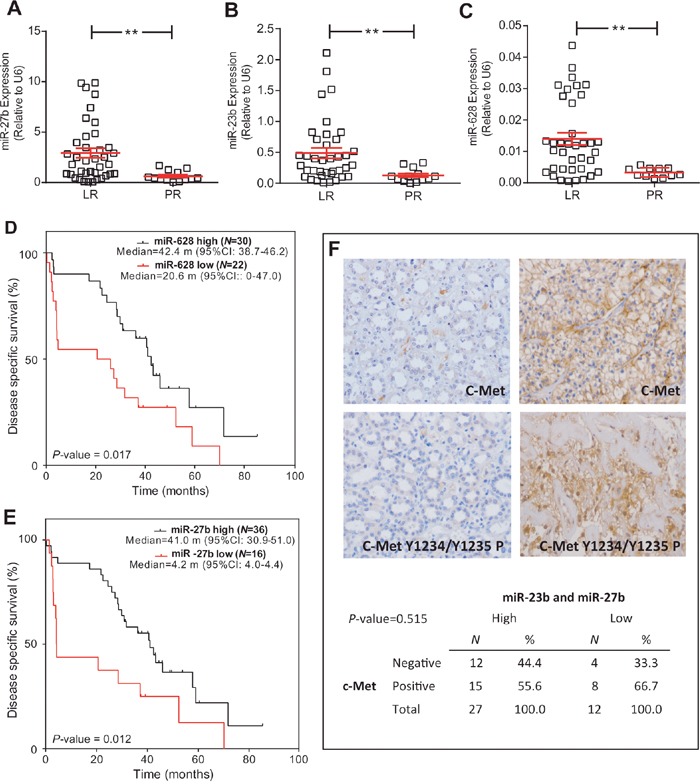
miRNA expression is associated with LR population **(A-C)** RTqPCR analyses showing the expression of miR-23b **(A)**, miR-27b **(B)** and miR-628-5p **(C)** in LR and PR populations. Statistical significance was obtained by Mann–Whitney's test ** *P*-value ≤0.01. **(D, E)** Kaplan-Meier analysis showing that high levels of miR-628-5p **(D)** and miR-27b **(E)** were associated with increased survival (p value was obtained by the log-rank test). **(F)** Representative immunohistochemistry images of positive and negative tumors for total C-Met and phosphorylated (in Tyr1234 and Tyr1235) c-Met, and contingency tables showing the absence of significant association between high levels of miR-23b and miR-27b and reduced c-Met staining. RTqPCR: Quantitative real-time reverse transcription polymerase chain reaction; LR: long-term responders; PR: primary refractory; CI: confidence intervals; m: months.

**Figure 4:**
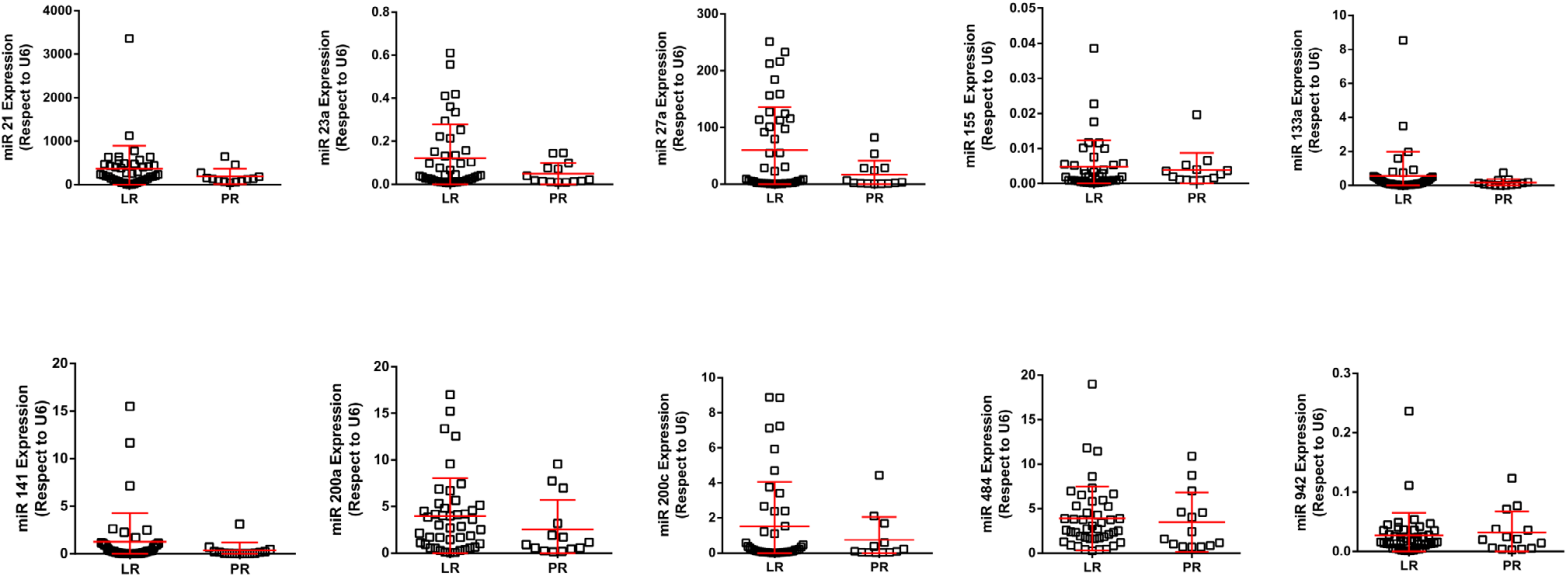
RTqPCR analyses showing the expression of quoted miRNAs in LR and PR populations RTqPCR: Quantitative real-time reverse transcription polymerase chain reaction; LR: long-term responders; PR: primary refractory.

Increased levels of 3 out of 13 studied miRNA were found in the LR population: hsa-miR-27b, hsa-miR-23b and hsa-miR-628-5p (Figure 3A, 3B, 3C; Figure 4). Additionally, the increased expression of these miRNAs was also associated with prolonged survival (AUC [ROC]=0.799; 0.793 and 0.800, respectively; cut-off values: 0.60; 0.15 and 0.005, respectively) (Figure 3D, 3E, and data not shown).

No validated targets have been yet identified for hsa-miR-628-5p. Both hsa-miR-27b and hsa-miR-23b inhibit the expression of c-Met and Notch1. A tendency of decreased c-Met activation in the LR group was observed, although statistical significance was not reached. Moreover, we did not find any association between increased levels of hsa-miR-23b or hsa-miR-27b, or the reduced levels of both, and the expression of c-Met (Figure 3F).

### Correlation between PFS and toxicity in LR group

**Figure 5:**
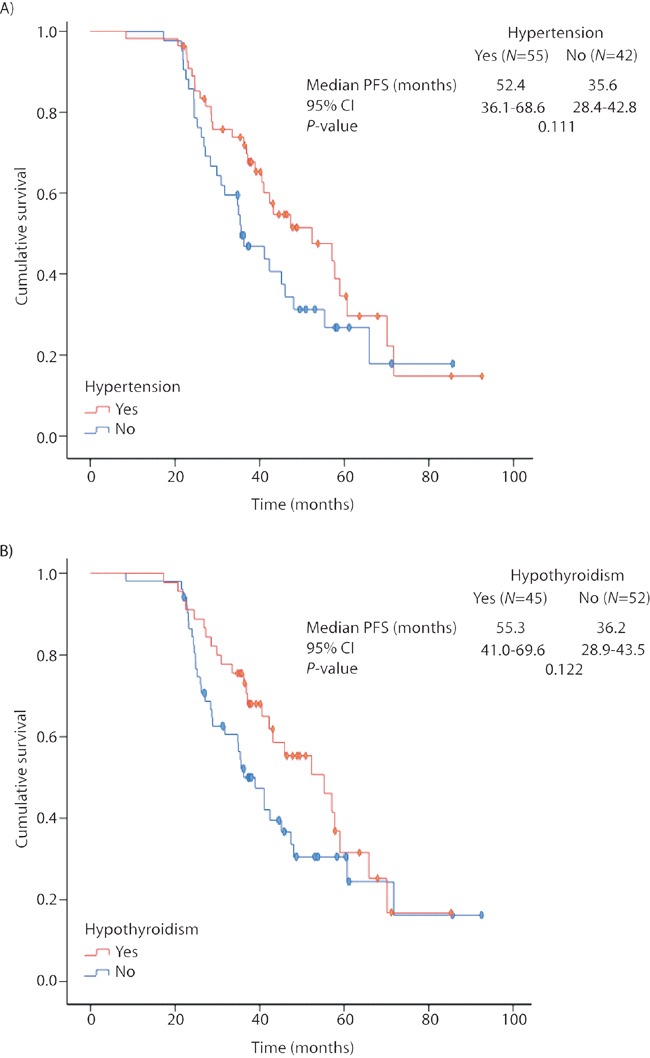
Progression-free survival in the long-term responders' cohort according to the occurrence of **(A)** hypertension; **(B)** hypothyroidism. Kaplan-Meier estimates. PFS: progression-free survival; CI: confidence intervals.

Ninety-four percent of LR patients presented at least one AE during the study, being the most common (all grades): asthenia (83%), mucositis (75%) and hand-foot syndrome (64%), followed by hypertension, hypothyroidism, neutropenia and thrombocytopenia (57%, 46%, 43% and 42%, respectively). A marked tendency towards prolonged PFS was observed in patients with hypertension or hypothyroidism, but did not reach statistical significance (Figure 5).

## DISCUSSION

The results of this molecular study suggest that there is a potential correlation between Notch activation and long-term response in mRCC patients treated with sunitinib. This correlation has been previously reported [17] and is aligned with the widely reported role of this pathway in angiogenesis [18, 19]. Our data also support that other relevant signaling pathways, such as Hedgehog and hypoxia, are not major players in sunitinib response in mRCC.

According to our analyses, only *HEYL* expression could be potentially used a biomarker of sunitinib response. Increased *HEYL* expression has been associated with neovascularization in breast cancer [20]. Nonetheless, other potential Notch signaling effectors, such as *HES1* and *HEY*, did not reach the statistical significance as unique biomarkers. Moreover, *JAG1* levels did not discriminated studied populations, and Notch IC was not associated with increased *HEYL* levels. In our opinion, the low number of available tumor samples and the high intratumor heterogeneity in mCRR [21] may be limiting these results. Notch signaling is a complex process involving multiple cell compartments and proteins, and its possible role in sunitinib response requires further investigation in a larger cohort.

Various miRNAs can regulate hypoxia and angiogenesis [22], suggesting a potential role in patient's response to antiangiogenic drugs. For instance, hsa-miR-221/222, hsa-miR-141, hsa-miR-942, hsa-miR-133a and hsa-miR-484 have been previously suggested as candidates for poor response to sunitinib [23–26]. However, there are important discrepancies among these studies, which are also limited by the small number of patients included and their specific clinical and therapeutic response characteristics. The results herein presented, comparing extreme groups of responders to sunitinib, do not support the potential significance of these pre-specified miRNAs. Here we provide evidence that changes in hsa-miR-628-5p are distinctive of LR and PR populations of sunitinib-treated mRCC patients. This miRNA was previously identified by Prior et al [25]. Nonetheless, the absence of bona fide characterized targets of hsa-miR-628-5p precludes ulterior functional analysis.

To the best of our knowledge, this is the first report showing an association between increased levels of hsa-miR-23b and hsa-miR-27b and prolonged response to sunitinib in patients with mRCC. However, their roles in other cancer types and in chemoresistance have been previously reported [27, 28]. The joint inhibition of the expression of Notch1 and c-Met by hsa-miR-23b and hsa-miR-27b reinforces our above commented hypothesis regarding the potential involvement of Notch signaling in sunitinib response, and warrants future research on this pathway. Furthermore, MET expression has been associated with resistance to sunitinib and with more aggressive tumor behavior in renal cancer, becoming an attractive target for the treatment of sunitinib-resistant mRCC patients [29]. However, we did not find a significant association between increased levels of hsa-miR-23b or hsa-miR-27b or the reduced levels of either miRNAs with the expression or activity of MET in renal cancer samples. Further studies are needed to elucidate the possible targets of these two miRNAs regarding sunitinib response in mRCC patients.

Additionally, despite the strong tendency observed, we did not found a significantly prolonged PFS in our cohort of LR patients presenting sunitinib-related AEs, contrary to previous studies [6, 7], probably due to their extremely prolonged survival time.

We observed a significantly higher number of Heng risk factors [30] in PR patients, although risk categories did not differ between groups, probably related with the low number of patients included in PR group. Additionally, we found a significantly lower proportion of nephrectomies, higher lactate dehydrogenase levels, shorter time to metastatic disease, higher presence of lung, brain and hepatic metastasis, and increased Fuhrman grade among PR patients. Some of these factors have been previously identified as predictive of a shorter PFS [14, 16], while others may have arose given the extreme differences of the studied populations. The validation of its prognostic value in a large independent cohort is warranted.

In conclusion, our data present important molecular implications regarding the response to sunitinib in mRCC patients. However, given the lack of identified molecular targets and current limitations of immunohistochemistry techniques, we were unable to identify which pathways are conditioning sunitinib response. The identification of these pathways and the confirmation of *HEYL*, hsa-miR-27b, hsa-miR-23b and hsa-miR-628-5p as predictive biomarkers will allow clinicians to offer an accurate and personalized treatment for both newly diagnosed and non-responder patients in daily clinical practice.

## PATIENTS AND METHODS

### Patients

Eligibility criteria included adult (age ≥18 years) patients with confirmed mRCC with a clear-cell component treated with sunitinib. Patients were classified in two groups:

- Long-term responders: patients who achieved a PFS ≥22 months and at least complete response (CR), partial response or stable disease (SD).
- Primary refractory: patients who showed progressive disease in the first 3 months after sunitinib onset.

### Study design and assessments

Retrospective, observational, multicenter study of two extreme groups of patients (LR and PR) treated with sunitinib under clinical practice. The study was conducted between January 2012 and January 2014 in 16 centers participating in the Spanish Oncology Geniturinary Group (SOGUG). The objectives were to identify baseline clinical factors associated with long-term clinical benefit to sunitinib treatment and to correlate tumor expression of different signaling pathways components and the occurrence of treatment-related AEs with treatment associated survival.

This study was carried out in accordance with the declaration of Helsinki and approved by the Spanish Authorities and Ethics Committees of each participating hospital. All patients gave written informed consent.

### Study procedures

Immunohistochemistry and genetic analyses were carried out in available archived formalin-fixed paraffin embedded (FFPE) clear-cell primary tumor samples. Hematoxylin and eosin stained sections of the tumor samples were examined by a pathologist to confirm the diagnosis and estimated tumor content. These analyses were performed at the Molecular Laboratory of Hospital 12 de Octubre (Madrid, Spain).

The following patient characteristics and outcome data were collected using uniform data collection templates: Date of birth, gender, age at CRC diagnosis, date of first diagnosis of tumor disease, baseline TNM, baseline cancer stage, histological subtype, Furhman grade, comorbidities, nephrectomy (yes/no), nephrectomy type (total/partial), percentage of tumor necrosis, Karnofsky/ECOG performance status, baseline lactate dehydrogenase level, baseline corrected calcium level, baseline hemoglobin level, left ventricular ejection fraction, presence of thrombocytosis at baseline, presence of neutrophilia at baseline, baseline Heng risk factors, previous radiotherapy received (yes/no), number of metastatic locations, recurrence (metastatic) location and date, and treatments received (dates, type, doses, associated toxicity and response [PFS, time to progression and OS]).

### Quantitative real-time reverse transcription polymerase chain reaction (RT-qPCR)

Expression levels of 13 selected miRNAs (miR-942, miR-628-5p, miR-133a, miR-484, miR-141, miR-23a, miR-23b, miR-27b, miR-21, miR-200a, miR-200c, miR-141and miR-155) and mRNAs (*MYC, VHL, HIF1A, HIF2, GLI1, GLI2, BMP4, PTCH1, HES1, HEY, HEYL, AXIN2*, SUFU and JAG1 were monitored by RT-qPCR in both groups. The miRNA were selected on the basis of their involvement in angiogenesis, modulation of the Notch, Hedgehog and/or Wnt signaling pathways, or by being previously reported as biomarkers of Sunitinb response in renal cancer samples [23–26]. For the mRNA normalization six previously reported genes were assayed (*ACTB, TBP, GusB, GAPDH, PPIA1* and *RPS13*) and evaluated using three normalizer evaluation software (GeNorm, NormFinder and BestKeeper). The best normalizer gene was *GusB*, so the expression analyses were performed relative to this gene.

Total RNA was isolated from ten 10 μm sections of each tumor sample and using miRNeasy FFPE Kit (Qiagen, Hilden, Germany) according to the manufacturer's recommendations. Reverse transcription was performed using the Omniscript RT Kit (Qiagen, Hilden, Germany), using 50 ng of total RNA and a mix of 10 mM of all specific RT primers (Table 2) in 50 μl final volume. Polymerase chain reaction (PCR) was performed in a 7500 Fast Real Time PCR System using Go Taq PCR master mix (Promega, Fitchburg, WI, USA) and 1 μl of cDNA as a template. Melting curves were performed to verify specificity and absence of primer dimers. Reaction efficiency was calculated for each primer combination. The sequences of the specific qPCR oligonucleotides are shown in Table 2. To measure quantitatively the expression of miRNAs, RNA was extracted using the same method as for the genes. Reverse transcription was carried out from 10 ng total RNA along with miR-specific primer using the TaqMan^®^ MicroRNA Reverse Transcription Kit (Applied Biosystems, Foster City, CA, USA). PCR assays were performed using TaqMan^®^ Gene Expression Master Mix and 7500 Fast Real Time PCR System (Applied Biosystems, Foster City, CA, USA) as reported [31]. For miRNA normalization, we used RNU6B. The potential genes targeted by the studied miRNA were assessed using the miRTarBase webtool (http://mirtarbase.mbc.nctu.edu.tw/) [32].

**Table 2:** Sequences of the specific RT primer oligonucleotides used in the reverse transcription polymerase chain reaction

GENE	RT	Forward	Reverse
*Hu-cMyc*	GTTGAAGGAATCG	AATGAAAAGGCCCCCAAGGTAGTTATCC	GTCGTTTCCGCAACAAGTCCTCTTC
*Hu-ACTB*	GCATTACATAATTTACAC	CCAACCGCGAGAAGATGA	TCCATCACGATGCCAGTG
*Hu-TBP*	GTG TTT AAA ATC TAC ATA	AGTGAAGAACAGTCCAGACTG	CCAGGAAATAACTCTGGCTCAT
*Hu-GUSB*	CTTCTGATACTTCTTATAC	CGCCCTGCCTATCTGTATTC	TCCCCACAGGGAGTGTGTAG
*Hu-GAPDH*	TACTTTATTGATGGTACA	AGCCACATCGCTCAGACAC	GCCCAATACGACCAAATCC
*Hu-PPIA1*	AATGGTGATTCTTCTTGCTGG	ATGCTGGACCCAACACAAAT	TCTTTCACTTTGCCAAACACC
*Hu-RPS13*	CTTAATTAAATGGTAGAGATC	GGTTGAAGTTGACATCTGACGA	CTTGTGCAACACCATGTGAAT
*Hu-VHL*	CCCTGACTGACTGAAGGCT	ATCCGTAGCGGTTGGTGA	CTCACGGATGCCTCAGTCTT
*Hu-Hif1a*	CTGCATGATCGTC	TTTTTCAAGCAGTAGGAATTGGA	GTGATGTAGTAGCTGCATGATCG
*Hu-Hif2*	TAGGTGAACTTCATGTCC	TACAAGGAGCCCCTGCTGTC	TGCTGGATTGGTTCACACATG
*Hu-Gli1*	TGACTTCTGTCCCCACACTG	AGCGCCCAGACAGAGTGT	GGGGTCATCGAGTTGAACAT
*Hu-Gli2*	AGCTGGCTCAGCATGGTC	ACTCCACACACGCGGAAC	CCACTGAAGTTTTCCAGG
*Hu-BMP4*	TCCACAGCACTGGTCTTGAG	TGGGATGTTCTCCAGATGTTCT	GGGATGCTGCTGAGGTTAAA
*Hu-PTCH1*	CGAGGTTCGCTGCTTTTAAT	TCTGGAGCAGATTTCCAAGG	TTTGAATGTAACAACCCAGTTTAAATA
*Hu-HES1*	GTGCGCACCTCGGTATTAAC	GAAGCACCTCCGGAACCT	GTCACCTCGTTCATGCACTC
*Hu-HEY*	AGCAGATCCCTGCTTCTCAA	CGAGCTGGACGAGACCAT	GGAACCTAGAGCCGAACTCA
*Hu-HEYL*	GGGCATCAAAGAATCCTGTC	GTCCCCACTGCCTTTGAG	ACCGTCATCTGCAAGACCTC
*Hu-AXIN2*	CTTCATCCTCTCGGATCTGC	GCTGACGGATGATTCCATGT	ACTGCCCACACGATAAGGAG
*Hu-SUFU*	ACTGCAGGGCCCA	TGTTGGAGGATTTAGAAGATTTGAC	AGGCCAGCTGTACTCTTTGG
*Hu-JAG1*	TTGATCATGCCCGA	GGCAACACCTTCAACCTCA	GCCTCCACAAGCAACGTATAG

### Construction of tissue microarrays (TMA) and immunohistochemistry

The expression of Notch IC (MAB3647 R&D Systems, Minneapolis, MN, USA; 1/100 diluted), HEYL (TA324613, Origene 1/500 diluted), c-MET (AF276, R&D Systems; 1/100 diluted) and c-MET phosphorylated in Y1234/Y1235 (AF2480, R&D Systems, 1/100 diluted) were assessed by immunohistochemistry in TMA after antigen retrieval treatment. Signal was amplified using avidin-peroxidase (ABC elite kit Vector, Burlingame, CA, USA) and visualized using diaminobenzidine as a substrate (DAB kit Vector, Burlingame, CA, USA). Scoring of the results and selection of the thresholds, internal controls for reactivity of each antibody, and tissue controls for the series were done by double blind method according previously published methods [33]. At least two representative duplicate cores for each case were scored.

### Statistical analysis

Protein, mRNA and miRNA expression (positive vs negative) were correlated with treatment outcome according to the cohorts of LR and PR. Survival probabilities were assessed by the Kaplan-Meier method. Cox proportional hazard models were used to identify predictors of treatment outcomes. To determine their ability to discriminate the two populations we sequentially performed unpaired tTest, ROC analyses and Kaplan-Meyer distributions with LogRank test. For all statistical tests, an a priori significant level of α=0.05 was assumed.

Efficacy end points included ORR, OS and PFS, assessed by investigators using Response Evaluation Criteria in Solid Tumours v1.1 [34]. The AEs were recorded regularly and graded according to Common Terminology Criteria for Adverse Events (CTCAE) v4.0. SPSS Statistics^©^ software version 19 (IBM, Armonk, NY) was used for statistical analysis.
